# Transduodenal ampullectomy provides a less invasive technique to cure early ampullary cancer

**DOI:** 10.1186/s12893-016-0156-z

**Published:** 2016-06-01

**Authors:** Yang Gao, Yayun Zhu, Xiuyan Huang, Hongcheng Wang, Xinyu Huang, Zhou Yuan

**Affiliations:** Department of General Surgery, Shanghai Jiao Tong University Affiliated Sixth People’s Hospital, No.600, Yishan Road, Shanghai, 200233 China

**Keywords:** Transduodenal ampullectomy, Pancreatoduodenectomy, Lymph node metastasis, Early ampullary cancer, Free resection margin

## Abstract

**Background:**

The aim of this study was to evaluate the clinical efficiency of transduodenal ampullectomy (TDA) compared to conventional pancreatoduodenectomy (PD) in patients with early ampullary cancers.

**Methods:**

We carried out a retrospective study by reviewing the medical records of 43 patients with early ampullary cancer who underwent either TDA or PD from January 2001 to December 2014. TDA and PD were performed on 22 patients and 21 patients, respectively. Clinical data, perioperative clinical outcomes and prognosis were evaluated. The median follow-up was 75 (range, 38–143) months.

**Results:**

The sensitivity of intraoperative frozen resection was 100 % (4/4) and 94.9 % (37/39) in patients with pTis and pT1 tumors compared to final histologic diagnoses. The 5-year survival rate of patients with early ampullary cancer was 77.3 % in TDA group and 75.9 % in PD group (*P* = 0.927). Patients with lymph node metastasis presented a shorter 5-year survival rate (*P* = 0.014). TDA was associated with lower surgical morbidity (*P* = 0.033), estimated blood loss (*P* = 0.002), medical cost (*P* = 0.028) compared to PD. No pancreatic fistula and surgical mortality occurred in TDA group.

**Conclusions:**

TDA could produce satisfactory clinical efficiency in patients fulfilled the following criteria simultaneously: pTis or pT1 stage, tumor size ≤ 2 cm, without lymph node metastasis. To achieve favorable outcomes, intraoperative frozen section examinations should be reliable and resection margins should be negative.

## Background

Ampullary cancer is an exceptionally uncommon gastrointestinal tumor with an incidence of 0.4–0.5 per 100,000 per year, which accounts for about 5 % of all gastrointestinal cancer diagnosed every year [1, 2]. It’s the second most common cancer after pancreatic cancer in the periampullary region and accounts for about 6–20 % of periampullary tumors [3]. It has been reported that ampullary cancer could be developed from adenomas by the adenoma-carcinoma progress similar to the development of colorectal cancer, which could be supported by the fact that up to 50 % of ampullary villous tumors harbored local adenocarcinoma at the time of diagnose and 80 % of ampullary adenocarcinomas contain adenomatous tissue [4, 5]. Therefore, there is no doubt that both benign and malignant ampullary lesions should be resected if conditions permit [2].

Pancreatoduodenectomy (PD), or Whipple procedure, was once considered as the only choice for the management of both benign and malignant tumors of the ampulla and the radical procedure could achieve a 5-year survival rate of 59.8 % for various stages and 83.7 % for early ampullary cancers (pTis, pT1), respectively [6]. However, PD also brings about relatively high surgical morbidity (25–50 %) and quite worrisome surgical mortality (approximately 5 %) despite the improvement of surgical techniques [7–9]. Transduodenal ampullectomy (TDA) has been proposed for more than one century since 1899 and it has been readmitted in the treatment of early ampullary tumors recently [10, 11]. TDA is a less invasive and simple technique, which could potentially provide equivalent clinical outcomes for early ampullary tumors compared to radical PD, while the indications for this local ampullectomy are still controversial. Previous studies have demonstrated the risk factors which would have an impact for the criteria of performing TDA. Lymph node metastasis, lymphatic invasion, resection margin and depth of invasion were critical prognostic factors [6, 12]. Some other factors were also reported to be associated with the prognosis of ampullary tumors, including pancreas invasion, perineural invasion, pathological subtype, grade of differentiation, tumor budding and intraoperative transfusion [6, 11, 13]. To achieve satisfactory clinical outcomes of TDA, two criteria should be taken into consideration: no lymph node metastasis and negative resection margin [5, 6]. Based on previous studies, we performed a retrospective study on whether TDA would be of any benefit to selected patients with early ampullary cancers compared to PD.

## Methods

### Patients

This study was approved by the ethics committee of the Shanghai Jiao Tong University Affiliated Sixth People’s Hospital and was performed in accordant with the Declaration of Helsinki Principles. Informed consent was obtained from each patient. Between January 2001 and December 2014, medical records of 43 patients with early ampullary cancer admitted to the Shanghai Jiao Tong University Affiliated Sixth People’s Hospital were reviewed. Early ampullary tumor was defined as tumor that confined to mucosa and ampulla of Vater or sphincter of Oddi according to American Joint Committee on Cancer (AJCC) 2010 staging [14]. The qualified patients received either TDA or PD procedure and they didn’t receive radiotherapy or chemotherapy. Clinicopathological characteristics were obtained including demographics, clinical presentations and postoperative outcomes. Postoperative mortality was defined as in-hospital or 30-day death, as well as with the morbidity recorded. Pancreatic fistula was defined and graded according to the International Study Group recomandations [15]. Follow-up information was obtained by either telephone interviews or outpatient visits.

### Surgery

All patients who met the following criteria were considered as suitable for TDA: 1) no lymph node metastasis was detected by imaging examinations and intraoperative frozen resection biopsy; 2) depth of invasion was limited to Tis and T1; 3) lesions no more than 2 cm. Although early ampullary cancer patients with systemic morbidities, such as pulmonary, cardiac and vascular disease, also received TDA, they weren’t included in this study to avoid confounding factors. If patients didn’t agree to receive this less invasive surgery, then PD procedure was performed. If patients didn’t fulfill the above criteria, conventional PD procedure was performed preferentially. Based on different surgical procedures, we categorized these patients into TDA group (*n* = 22) and PD group (*n* = 21).

**Fig. 1:**
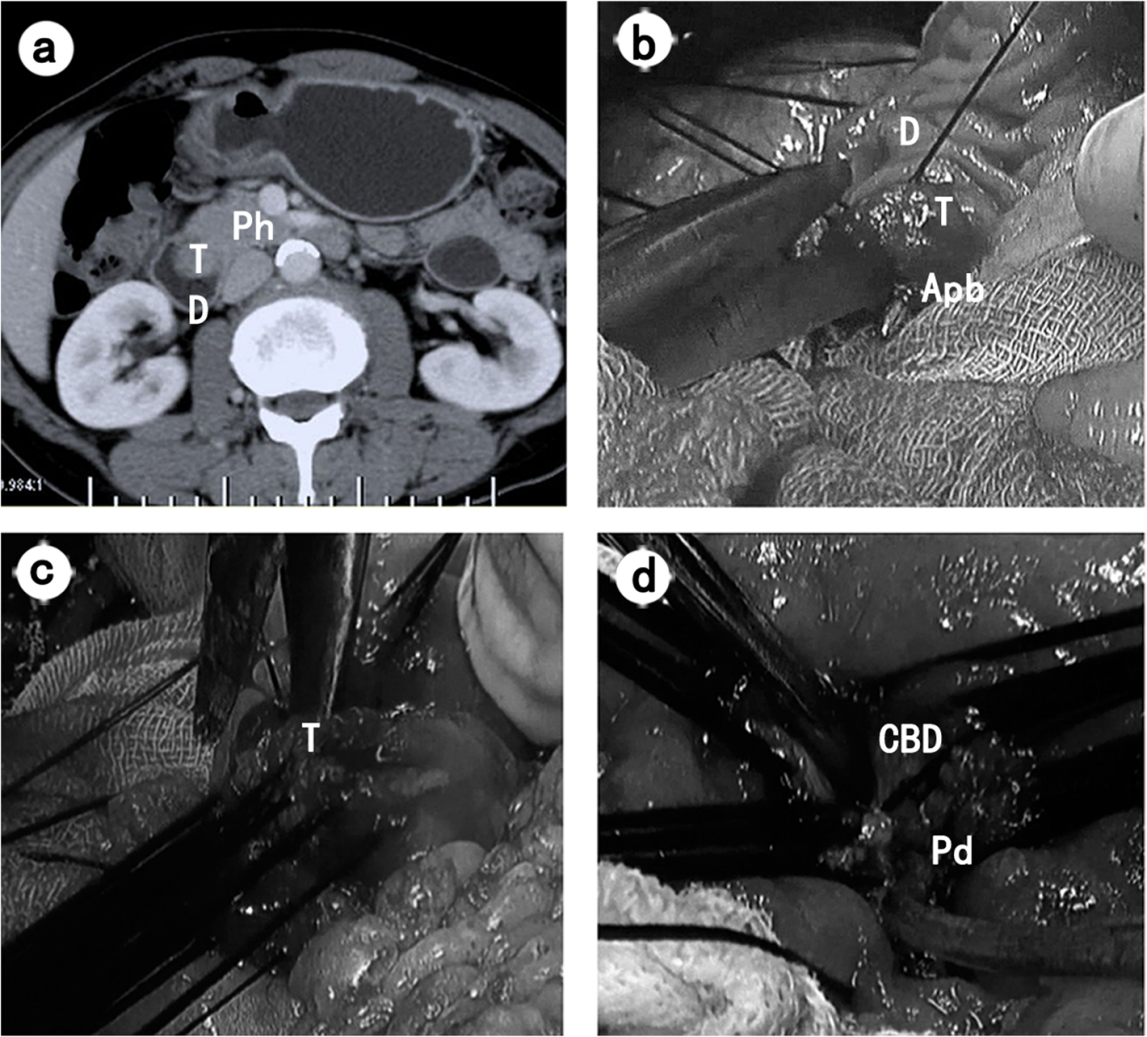
Main surgical technique of TDA. **a** Preoperative CT image showed the ampullary tumor protruded into duodenal lumen as a bulging. **b** Intraoperative photograph showed that local resection of the tumor started from the site of the 11 o’clock position. **c** Intraoperative photograph showed the circumferential resection of the tumor was about 5–10 mm from the edge of the tumor. **d** Intraoperative photograph showed the reconstructed ampullary region. *T* ampullary tumor; *D* duodenum; *Ph* pancreatic head; *Apb* ampullo-pancreatobiliary common duct; *CBD* common biliary duct; *Pd* pancreatic duct

Surgical technique of TDA has been described in previous literatures in detail [5, 16]. In short, after an upper midline incision, Kocher maneuver was then performed to mobilize the descending part of duodenum. Routine lymph node resection was performed and then sent for frozen pathological examination to confirm negative lymph node metastasis, including supraduodenal as well as anterior and posterior lymph nodes of the pancreatic head. After en block tumor was removed, both the tumor tissue and resection margin were sent for frozen pathological examination. If the results fulfilled the potential local resection criteria, then TDA continued; otherwise, the operation was converted to radical PD. Then, common bile duct and pancreatic duct were reconstructed by a reliable interrupted full-thickness suture. To reduce surgical morbidity, T-tube was placed in the common bile duct and its distant end reached to duodenal lumen and a rubber stent was placed into the pancreatic duct. The transverse suture was applied to close the duodenum to avoid duodenal stenosis. The main surgical process was demonstrated in Fig. 1. The surgical technique of PD has been described before [17, 18]. The pancreatic anastomosis was done in an end-to-end telescoped fashion into the jejunum. Routine intraoperative drains were performed.

### Statistical analysis

Statistical analysis was performed by SPSS 19.0 software (IBM SPSS Inc., Chicago, IL, USA). Survival was measured from the date of surgery and follow-ups were finished on the December 31, 2014. Categorical variables were compared using chi-square/Fisher’s exact test. Continuous variables were compared by *t* test. Survival rates were estimated by the Kaplan-Meier method, and log-rank test were used to analyze the difference. A *P* value of less than 0.05 was considered statistically significant.

## Results

### Demographics and clinical features

The demographics, clinical manifestations of 43 study subjects were summarized in Table 1. Of the 43 patients (24 male and 19 female) diagnosed as early tumor of the VA, 4 pTis tumors and 18 pT1 tumors were in TDA group and PD group only consisted of 21 pT1 tumors. No statistical difference was observed between TDA group and PD group regarding sex, age, clinical presentation, tumor size, carbohydrate antigen 19–9 (CA19-9), carcinoembryonic antigen (CEA). The mean age of the patients undergoing TDA and PD was 66.4 (range, 38–87) years and 64.7 (range, 36–83) years. The clinical presentations in patients undergoing TDA or PD were as follows, respectively: abdominal pain, 13 (59.1 %) and 10 (47.6 %); jaundice, 4 (18.2 %) and 5 (23.8 %); fever 5 (22.7 %) and 7 (33.3 %); nausea/vomiting, 3 (13.6 %) and 2 (9.5 %); asymptomatic, 4 (18.2 %) and 3 (14.3 %). Of the 7 asymptomatic patients, 5 were diagnosed by the computed tomography (CT) and 2 by magnetic resonance imaging (MRI). The mean tumor size in TDA group and PD group was 1.2 cm (range, 0.4–2.0 cm) and 1.2 cm (range, 0.6–2.3 cm). The lymph node metastasis was identified in 28.6 % (6/21) in the PD group and patients with lymph node metastasis are not qualified for TDA.

**Table 1:** Demographics, clinical presentations, and CAV characteristics

	TDA (*n* = 22)	PD (*n* = 21)	*P* value
Sex			0.545
Male	13 (59.1 %)	11 (52.4 %)	
Female	9 (40.9 %)	10 (47.6 %)	
Age (years)			0.722
Median, range	68.0 (38–87)	67.0(36–83)	
Symptom			
Abdominal pain	13 (59.1 %)	10 (47.6 %)	0.451
Jaundice	4 (18.2 %)	5 (23.8 %)	0.937
Fever	5 (22.7 %)	7 (33.3 %)	0.538
Nausea/vomiting	3 (13.6 %)	2 (9.5 %)	1.000
Asymptomatic	4 (18.2 %)	3 (14.3 %)	1.000
Tumor Size (cm)			0.725
Mean, range	1.2 (0.4–2.0)	1.2 (0.6–2.3)	
CA19-9 (U/ml)	3.20 ± 5.21	3.16 ± 2.16	0.957
CEA (ng/ml)	9.39 ± 5.21	12.07 ± 6.07	0.154
Depth of invasion			0.108
pTis	4 (18.2 %)	0 (0 %)	
pT1	18 (81.8 %)	21 (100 %)	
Lymph node metastasis			0.009
Positive	0 (0 %)	6 (28.6 %)	
Negative	22 (100 %)	15 (71.4 %)	

### Pathologic findings

The preoperative, intraoperative and final pathological examination results were depicted in Table 2. The preoperative endoscopic biopsy and intraoperative frozen resection have been routine clinical practice in the compound management of ampullary cancer in our department. A true positive result was determined by the final paraffin resections. When the final histologic diagnoses after TDA or PD procedure were compared with preoperative endoscopic biopsy, the sensitivity was 50 % (2/4) and 76.9 % (30/39) in patients with pTis and pT1 tumor, respectively. When the final histologic diagnoses after surgical treatment were compared with the intraoperative frozen resection, the sensitivity was 100 % (4/4) and 94.9 % (37/39) in patients with pTis and pT1 tumor, respectively. The false negative results were mainly due to misdiagnoses of chronic mucous inflammation and adenoma with various level of dysplasia. Notably, the negative margin (R0) resections were all achieved among the 43 patients according to the final pathology.

**Table 2:** Pathology on endoscopic biopsy, intraoperative frozen section, and final pathology

Final pathology	Endoscopic biopsy			Intraoperative frozen resection		
	Inflammation	Adenoma	Carcinoma	Inflammation	Adenoma	Carcinoma
pTis (*n* = 4)	1	1	2	0	0	4
pT1 (*n* = 39)	2	7	30	0	2	37

### Perioperative outcomes

In PD group, 1 of the 21 patients (4.7 %) died of pancreatic fistula and intractable sepsis 10 days after PD procedure. The surgical morbidity of PD group was significantly higher than that of TDA group (47.6 % vs. 18.2 %, *P* = 0.033). Pancreatic fistula, as a vital complication, only occurred in PD group with a quite high incidence rate of 19.0 %. Subsequently, the lengths of postoperative stay were shorter in TDA group compared to PD group (14.5 ± 4.8 days vs. 19.0 ± 7.9 days, *P* = 0.029). Similarly, patients undergoing TDA could spend less medical cost than those undergoing PD (7974.8 ± 4523.3 US dollar vs. 10813.5 ± 3541.8 US dollar, *P* = 0.028). The recurrence rate in the TDA group tended to be higher compared with the PD group, but the statistical difference was not reached (31.8 % vs. 23.8 %, *P* = 0.588). The operation time in TDA group was significantly shorter than that in PD group (175 min vs. 315 min, *P* < 0.001). Estimated blood loss during surgery was lower in the TDA group than PD group (135 ml vs. 320 ml, *P* = 0.002) and intraoperative transfusion was performed in 4 patients in PD group, while no one in TDA group received transfusion (Table 3).

**Table 3:** Postoperative outcomes after TDA or PD

Outcomes	TDA (*n* = 22)	PD (*n* = 21)	*P*-value
Surgical mortality,%	0 (0 %)	1 (4.8 %)	0.488
Surgical morbidity,%	3 (13.6 %)	9 (42.8 %)	0.033
Pancreatic fistula	0 (0 %)	4 (19.0 %)	0.048
Wound infection	2 (9.1 %)	3 (14.3 %)	0.664
Bleeding	1 (4.5 %)	5 (23.8 %)	0.082
Lengths of stay, days (mean, SD)	14.5 ± 4.8	19.0 ± 7.9	0.029
Medical cost, US dollar	7974.8 ± 4523.3	10813.5 ± 3541.8	0.028
Recurrence rate	7 (31.8 %)	5 (23.8 %)	0.558
Estimated blood loss (ml) (median, range)	135 (60–370)	320 (120–1800)	0.002
Intraoperative transfusion	0	4 (19.0 %)	0.048
Operation time (min) (median, range)	175 (122–269)	315 (233–389)	<0.001

### Prognosis after surgical treatments

**Fig. 2:**
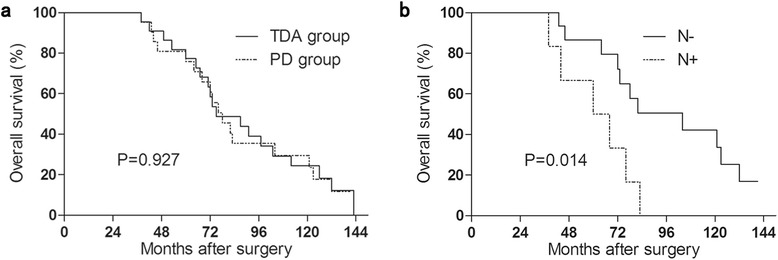
Kaplan-Meier plot. **a** Survival curve between TDA group and PD group, survive rate was calculated by the Kaplan-Meier method, significant difference wasn’t found between the two groups (*P* = 0.927). **b** Overall survival rate in PD group according to the lymph node status, patients without LN metastasis (N-)had better prognosis than these with LN metastasis (N+) (*P* = 0.014)

The median follow-up period was 75 (range, 38–143) months. The 5-year survival rate of the early ampullary in TDA group and PD group was 77.3 % (median survival time, 75 months) and 75.9 % (median survival time, 78 months) separately, which wasn’t statistically different (*P* = 0.927) (Fig. 2a). Furthermore, the 5-year survival rate of the whole 43 patients was 76.7 %. Regarding to surgical procedures, in TDA group, patients with pTis and pT1 showed 5-year survival rate of 100 % and 72.2 %, yet the difference didn’t reach statistical significance (*P* = 0.928). Patients in PD group were all at pT1 stage. In order to exclude the impact of depth of invasion and lymph node metastasis, 4 pTis patients in TDA group and 6 patients with LN metastasis in PD group were removed and the 5-year survival rate of the patients with pT1 undergoing TDA or PD were still not significantly different (*P* = 0.545).

Lymph node (LN) metastasis was a critical predictive factor for the prognosis of ampullary cancer, so we further investigate the overall survival and LN metastasis. Of the 21 patients receiving the PD procedure, 6 (28.6 %) patients had lymph node metastasis based on hematoxylin-eosin staining and histological examinations. The 5-year survival rate of patients in the PD group with LN metastasis was significantly lower than that in the patients without LN metastasis (*P* = 0.014) (Fig. 2b).

## Discussion

The surgical treatments for ampullary cancer mainly include PD and TDA. PD is the standard surgical strategy and TDA only accounts for about 4–6 % of cases of resected ampullary tumors [1, 19]. Endoscopic ampullectomy is another choice for ampullary lesions, but it is only suitable for low-grade and high-grade dysplasia, minor papilla adenoma [8, 20, 21]. For malignant ampullary tumors, endoscopic ampullectomy is not recommended due to the difficulty in the exact diagnose of early ampullary tumors, potential lymph node metastasis and significant complications [22]. TDA is a less invasive procedure compared to PD and it is likely to provide similar clinical outcomes for selected early ampullary cancer patients [23, 24]. However, the indications and clinical outcomes of TDA procedure still require further investigation. In this study, TDA group have similar 5-year survival rate and recurrence rate, but lower surgical morbidity, estimated blood loss, intraoperative transfusion and operation time compared with PD group, so the clinical value of TDA should be reconsidered. Although T stage and LN metastasis were independent prognostic factors, due to the small simple of patients with pTis and LN metastasis, no significant difference was found in the two groups. Specifically, when 5-year survival rate of pT1 patients without LN metastasis in TDA group and PD group were compared, there was still no significant difference, thus further implying that the equivalent clinical efficiency of TDA procedure in this part of patients.

Lymph node metastasis was a major factor for postoperative recurrence and overall survival [24, 25]. In our series, the 5-year survival rate in early ampullary tumors with and without lymph node metastasis was approximately 50 % and 80 %, respectively. In T1 tumors, lymph node metastasis is reported to decrease 5-year survival rate from about 75 to 30 % [25, 26]. Besides, the 3-year recurrence rate was significantly higher in patients with lymph node metastasis compared to those without (38.2 % vs. 58.78 %) [25]. In order to achieve operative curability in TDA procedure, lymph node metastasis shouldn’t appear in the selected cases [6]. Negative lymph node metastasis is essential for curative resection for ampullary cancer. Since pTis tumor is only confined to the mucosa, so there is no potential lymph node metastasis and this phenomenon has been confirmed by many studies. [2, 11]. In this regard, pTis tumor is appropriate indication for local ampullectomy. However, pT1 tumor has invaded to Sphincter of Oddi or ampulla of Vater, so lymph node metastasis usually occurred in 9–45.5 % of T1 tumors [11]. Masato Kayahara et al. reported that the most important lymph node was posterior pancreaticoduodenal lymph nodes and lymph nodes around superior mesenteric artery, which occurred in 39 % and 17 % of 36 ampullary cancer patients in 1997, which could be regarded as sentimental lymph nodes [26]. Afterwards, surgeons became to pay special attention on the anterior and posterior pancreatic lymph nodes and supraduodenal lymph nodes [5, 27]. In this study, we also perform supraduodenal as well as anterior and posterior lymph nodes of the pancreatic head and resulted in a comparable high clinical outcome [13]. Therefore, it’s of utmost importance to identify lymph node status before performing TDA.

Preoperative endoscopic biopsy is also routinely performed with a diagnostic accuracy of 69–81 % [2]. Biopsies should be taken in 9 to 1 o’clock quadrant to avoid inducing pancreatitis [8]*.* If the endoscopic biopsy showed benign lesions, then TDA could be considered. Because false-negative rate could be 10–38 %, so the possibility of malignance couldn’t be excluded [2]. When the results showed malignant tumors, more parameters, including tumor size, depth of invasion and lymph node metastasis should be considered if TDA was about to be performed. When the size of tumor was no more than 2 cm, the depth of invasion was pTis or pT1 and there was no sign of lymph node metastasis, TDA still could be considered [5]. Compared to the limited accuracy of endoscopic biopsy, the intraoperative biopsy could differentiate benign and malignant tumors with a sensitivity of 97 % and specificity of 100 % [28]. In addition, frozen section examination during ampullectomy helped obtain free resection margin and confirm the lymph node status [5]. In this study, the sensitivity of frozen resection biopsy was 100 % and 94.9 % for pTis and pT1 tumors, which could guarantee reasonable managements.

Although tumor size was not related to the presence or absence of malignancy and proximal ductal invasion, lymph node metastasis tended to occur more frequently with the size of tumor increasing [6, 10, 29]. Therefore, only when the size of tumor was less than 2 cm, TDA was performed. Since the small sample size of the patients with postoperative occurrence, we could hardly draw meaningful conclusions on the relationship between tumor size and recurrence, so large-scale clinical experiment should be designed to explore this issue. However, previous study may cast light on the problem. Yoo-Seok Yoon et al. has demonstrated that tumor size less than 1.0 cm, 1.1–1.5 cm, 1.6–2.0 cm showed lymph node metastasis rates of 11.6, 25.8, and 43.2 % [6]. In addition, lymph node metastasis was an independent risk factor for prognosis, therefore, we speculate that the smaller tumor size may be associated with lower recurrence rate [6, 25]. In terms of R0 resection, all of the cases in our series achieved the complete resection which contributed to improved 5-year survival, and the predictive value wasn’t analyzed. However, other studies have confirmed the prominent significance of R0 resection. Beger et al. reported that patients with R0 resection had significant superior survival comparing to these with R1 and R2 resection and R0 resection proved to be one critical prognostic factor [27]. It’s mandatory to ensure R0 resection for achieving long-term survival and we recommended that the resection should be 5–10 mm from the edge of the tumor if possible.

As to adjuvant chemoradiotherapy, on the one hand, adjuvant chemoradiotherapy couldn’t significantly prolong overall survival and reduce recurrence rate, so routine use of adjuvant chemoradiotherapy is not warranted [30, 31]. On the other hand, the patients in this study were early ampulllary tumors, while chemoradiotherapy might only benefit some patients with ampullary tumors with more invasive features [32]. Therefore, adjuvant chemoradiotherapy was not administered on this subset of patients. Additionally, local recurrence was not significantly different in TDA group and PD group. This result could be accounted by the fact that adequate free resection margin and negative lymph node metastasis was important for lower local recurrence [5, 25].

There are several limitations in this study. Since the technique of TDA is not quite popular and indications for this operation is not quite very obvious, study sample size is small and this study has to take the 15 years’ experience into consideration to produce significant statistical power. However, since the operations were performed in a single center and the same team, the surgical technique was quite stable, thus minimizing the confounder confounding factors. With the development of surgical technique and clinical study, this operation may be more popular and more cases will be available for further study. On the other hand, because it is a respective study, there exists selection bias and information bias, thus leading to less strong evidence. Therefore, there is an urgent need for prospective study by collaborating among multiple centers for exploring proper indications and treatment regimens.

In general, the perioperative clinical outcomes in TDA group were more favorable than these in PD group. Firstly, there was no surgical mortality in TDA group but 1 patient died of pancreatic fistula and intractable sepsis in PD group, which was quite troublesome. Although the surgical mortality of PD procedure has decreased to 5 % in high-volume hospitals, the surgical mortality does exist and how to avoid the mortality is very crucial [33]. Secondly, the surgical morbidity length of stay in TDA group was significantly lower compared to PD group and what’s more, pancreatic fistula didn’t occur in TDA group but occurred in 19 % of patients in PD group. Pancreatic fistula was a serious surgical morbidity and occurred in 5–30 % of patients, which could result in intra-abdominal abscess, sepsis, and even death [34]. Therefore, pancreatic fistula was a great challenge that waited to be solved or circumvented by hepatopancreaticobiliary surgeon. Thirdly, blood loss was much less and no intraoperative transfusion was needed in TDA group; in contrast, 19.0 % of patients received transfusion in PD group. Since intraoperative transfusion was associated with recurrence and shorter survival, the unnecessary blood transfusion should be avoided [35, 36]. Lastly but not least, medical cost in TDA group was lower than that in PD group due to lower and milder surgical morbidity and shorter length of stay, which will also be beneficial to patients.

## Conclusion

The TDA procedure is suitable for ampullary cancer patients fulfilled the following criteria simultaneously: pTis or pT1 stage, tumor size ≤ 2 cm, without lymph node metastasis. TDA procedure is feasible for selected patients with similar 5-year survival rate and lower surgical complications. Notably, TDA procedure should be based on reliable endoscopic biopsy, intraoperative frozen section examinations, and negative resection margins. Due to the number of patients was small in this study, more clinical studies should be carried out to validate the safety and efficiency of the TDA in qualified patients.

## Abbreviations

Apb, ampullo-pancreatobiliary common duct; CA19-9, carbohydrate antigen 19–9; CBD, common biliary duct; CEA, carcinoembryonic antigen; CT, computed tomography; D duodenum; LN, lymph node; MRI: magnetic resonance imaging; Pd, pancreatic duct; PD, pancreatoduodenectomy; Ph, pancreatic head; T, ampullary tumor; TDA, transduodenal ampullectomy.
